# Ionomic and metabolic responses to neutral salt or alkaline salt stresses in maize (*Zea mays* L.) seedlings

**DOI:** 10.1186/s12870-017-0994-6

**Published:** 2017-02-10

**Authors:** Rui Guo, LianXuan Shi, Changrong Yan, Xiuli Zhong, FengXue Gu, Qi Liu, Xu Xia, Haoru Li

**Affiliations:** 1 Institute of Environment and Sustainable Development in Agriculture (IEDA), Chinese Academy of Agricultural Sciences (CAAS) / Key Laboratory of Dryland Agriculture, Ministry of Agriculture, Beijing, 100081 People’s Republic of China; 2 Key laboratory of Molecular Epigenetics of Ministry of Education (MOE), Northeast Normal University, Changchun, 130024 China

**Keywords:** Maize, Neutral salt stress, Alkaline salt stress, Growth, Photosynthesis, Metal elements, Metabolites

## Abstract

**Background:**

Soil salinity and alkalinity present a serious threat to global agriculture. However, most of the studies have focused on neutral salt stress, and the information on the metabolic responses of plants to alkaline salt stress is limited. This investigation aimed at determining the influence of neutral salt and alkaline salt stresses on the content of metal elements and metabolites in maize plant tissues, by using mixtures of various proportions of NaCl, NaHCO_3_, Na_2_SO_4_, and Na_2_CO_3_.

**Results:**

We found that alkaline salt stress suppressed more pronouncedly the photosynthesis and growth of maize plants than salinity stress. Under alkaline salt stress conditions, metal ions formed massive precipitates, which ultimately reduced plant nutrient availability. On the other hand, high neutral salt stress induced metabolic changes in the direction of gluconeogenesis leading to the enhanced formation of sugars as a reaction contributing to the mitigation of osmotic stress. Thus, the active synthesis of sugars in shoots was essential to the development of salt tolerance. However, the alkaline salt stress conditions characterized by elevated pH values suppressed substantially the levels of photosynthesis, N metabolism, glycolysis, and the production of sugars and amino acids.

**Conclusions:**

These results indicate the presence of different defensive mechanisms responsible for the plant responses to neutral salt and alkaline salt stresses. In addition, the increased concentration of organic acids and enhanced metabolic energy might be potential major factors that can contribute to the maintenance intracellular ion balance in maize plants and counteract the negative effects of high pH under alkaline salt stress.

## Background

At present, soil salinization is a serious environmental problem, seriously affecting global agriculture and exerts complex adverse effects on plant metabolism [1–3]. Na^+^, K^+^, Ca^2+^, Cl^−^, NO_3_^−^ and H_2_PO_4_^−^ are the predominant ions in naturally saline soils [1]. Despite the frequent coexistence of soil salinization and alkalization in the majority of cases, studies have mainly focused on salt stress and relatively little attention has been paid to alkaline stress [4–6]. The salt-alkaline stress, alkaline soils and calcareous soils with high pH value have also been investigated, but the information on alkaline stress is still scarce [7–9].

Previous researches have demonstrated that salt stress is caused by neutral salts, whereas alkaline stress is induced by alkaline salts [10–12]. By disrupting the ion homeostasis in plant cells, neutral salt stress leads to adverse osmotic conditions and damage caused by ions. However, although alkaline salt stress exerts the same negative effects, its adverse influence is further aggravated when it is combined with high pH value [13, 14]. There is evidence that the high pH in the rhizosphere lowers the availability of ions of nutrient elements, such as Ca^2+^, Mg^2+^, Cl^−^ and H_2_PO_4_^−^, by causing their precipitation [15]. Under the precipitated form, ion uptake is hindered, leading to disruption of ion homeostasis [16]. The high pH values can also have a direct detrimental effect on the structure of root cell membrane affecting substantially its structural functions [17]. Hence, to adapt to the conditions of an alkaline soil, plants should have the ability to endure ion toxicity and physiological drought, as well as to maintain their intracellular ion balance and counteract the negative pH changes outside roots. Plants can respond to alkaline salt stress by changes in certain metabolic processs that might be involved, such as photosynthesis, ion transport, synthesis of hormones, and accumulation of osmotic solutes [11, 16, 17]. The activities of some metabolic solutes, including betaine, proline, polyamine, and polyhydric alcohol, are beneficial to achieving tolerance to neutral salt stress [1, 14]. Moreover, certain metabolic compounds might also be involved in the tolerance of plants to high pH, but insufficient information is available on this potential adaptive mechanism [16, 17]. Therefore, a comprehensive metabolic analysis of the responses of plants to adverse high salinity and alkalinity conditions should be performed to identify the metabolic compounds that are associated with a response specifically elicited to high ion concentrations and high pH values. The determination of such metabolic components is also required to elucidate the mechanisms of plant tolerance to high salinity and alkalinity. Functional genomic research findings have complemented the results of metabolic analyses, revealing the specific responses of biosystems to environmental and genetic changes, including improvement in plant metabolome, comparison between laboratories and experiments, and enhancement of metabolomic data with other functional genomics information [18]. Metabolomic analyses have also been performed to elucidate the defensive mechanisms involved in plant tolerance and adaptation to neutral salt stress, including detoxification, ion homeostasis, compatible solutes synthesis and accumulation [1, 19–23].

Maize is a cereal crop that is grown widely throughout the world; it has adapted to various types of environment. Therefore, maize’s frequently used as a model crop to understand the respond to salinity conditions in the cereal crops [24–26]. In the present study, we systematically analyzed the metal elements and metabonomic features of maize plants to salt and alkaline salt stresses using ICP-OES and GC-MS in conjunction with multivariate data analysis. The primary purposes of such research are to investigate changes in the maize ionome in response to salt or alkaline salt stress; determine the possible difference in tissue ionome responses to neutral salt or alkaline salt stress; and define the metabonome of maize plants and metabolic profiles changes associated with neutral salt or alkaline salt stress as a function of salinity stress.

## Methods

### Plant materials and growing conditions

Seeds of Zhengdan-18, a salt-resistant variety of maize (*Zea mays* L.), which were kindly provided by the Crop Breeding Center of Chinese Academy of Agricultural Sciences. Seeds were immersed in de-ionized water for two days in a growth chamber (30 °C during the day and 25 °C at night). Then, a total of 100 seeds of maize were sown in 20 plastic pots with a length of 34 cm, width of 24 cm, and height of 12.5 cm (five seeds in each pot) that each of which was filled with 5.5 kg of washed sand. The experiments was carried out in late May to early July, all pots were placed outdoors and avoided rainfall. The temperatures during the experiment were 25 ± 2 °C during the day and 20 ± 2 °C at night. The resulting seedlings that grew in each pot were watered daily with sufficient quantities of 0.5 Hoagland’s nutrient solution [27].

### Design of simulated neutral salt or alkaline salt stress conditions

The salinity stress treatments used in this study were divided into neutral salt stress (NaCl and Na_2_SO_4_, at a 9:1 molar ratio) and alkaline salt stress (NaHCO_3_ and Na_2_CO_3_, at a 9:1 molar ratio). To induce neutral salt and alkaline salt stress, the maize seedlings were treated with two concentrations of each respective salt (50 and 100 mM). In the 100-mM solution used to cause neutral salt stress 90 mM NaCl and 10 mM Na_2_SO_4_ were mixed, achieving total ion concentrations of 110 mM Na^+^ + 90 mM Cl^−^ + 10 mM SO_4_^2−^. In the 100 mM solution used to cause alkaline salt stress, a mixture of 90 mM NaHCO_3_ and 10 mM Na_2_CO_3_ resulted in total ion concentrations of 110 mM Na^+^ + 90 mM HCO_3_^−^ + 10 mM CO_3_^2−^ (Table 1).

**Table 1:** pH, electrical conductivity (EC), and osmotic potential (OP) of the stress treatment solutions

Treatment	Salinity (mM)	pH	EC (dS m^−1^)	OP (Mpa)
Control (CK)	0	6.68	1.6	−0.05
Neutral salt stress (SS)	50	6.49	6.14	−0.25
	100	6.55	10.68	−0.56
Alkaline salt stress (AS)	50	8.86	5.27	−0.23
	100	9.26	8.89	−0.51

### Treatments

Four-week-old maize seedlings with uniform growth statuses grown in the 20 pots were divided randomly into four groups consisting of five pots. Each pot was considered a single replicate, and five replicates were presented in each group. One of the groups was untreated (control), which were watered with 0.5 Hoagland’s nutrient solution as usual. One group was used for the measurements of growth indices before salinity treatment. Last two groups were with either neutral salt stress or alkaline salt stress. The treatment groups were thoroughly watered daily at 17:00 to 18:00 to obtain the appropriate salinity stress conditions. Stress treatments lasted 15 days.

### Determination of photosynthetic and growth indices

The following formula was used to determine the relative growth rate (*RGR*): [ln dry weight after stress treatment − dry weight (DW) start stress treatment] / duration of treatment (days) [28]. Using a LI-6400XT Portable Photosynthesis System – (LI-COR Biosciences, Lincoln, NE, USA), we determined the net photosynthetic rate (*P*_n_), stomatal conductance (*g*_s_) and transpiration rate (*E*) that were investigated by performing daily measurements (in the morning, 10:00 am) at the first completely expanded leaf blade. Through the use of diodes emitting red-blue light sources, we subjected the seedlings to treatment with photosynthetically active radiation (1200 μmol m^−2^ s^−1^). Fresh leaves of plant material (100–150 mg) were treated with 10 ml of acetone for 2-4days in darkness, and the extract used to determine the contents of chlorophyll a and b, and carotenoids [29]. Each sample was then subjected to five-fold spectrophotometric analysis at wavelengths of 440 nm, 645 nm, and 663 nm, the calculations used the equations of Arnon: the contents of chlorophyll *a* = 9.784 × A_663_-0.99 × A_645_; the contents of chlorophyll *b* = 21.426 × A_663_-4.65 × A_645_; the contents of carotenoids = 4.695 × A_440_-0.268 × (Chl *a* + Chl *b*) [30].

### Measurement of metal elements

To determine the content of metal elements, we ground dried maize roots and shoots using a muffle furnace and subjected approximately 0.1 g of each tissue sample to thermal decomposition at 500 °C for 6 h. Further, we added 10 mL of HNO_3_:H_2_O_2_ (1:1) to each sample for extraction. The contents of metal elements were determined using an ICP-OES spectrometer (iCAP 6000 series, Thermo Fisher Scientific Inc.), according to the manufacturer’s manual. Analytical lines of ICP-OES were Na (589.0 nm), K (766.5 nm), Ca (402.6 nm), Mg (282.5 nm), Fe (238.2 nm), Cu (327.4 nm), Zn (213.9 nm), Mn (258.5 nm) and B (249.8 nm).

### Measurement of metabolites

Approximate 100 ± 3 mg of each tissue materials were placed in a centrifuge tube (2 mL). Next, 60 μl of ribitol (0.2 mg · ml^−1^ stock in H_2_O) was added to each tube, and 0.3 and 0.1 mL of methanol and chloroform were admixed with the samples by vortexing and grinding for 5 min in a mill system (70 Hz; Jinxin Biotech LTD., Shanghai, China). Samples were dissolved in 80 μL of methoxamine hydrochloride (20 mg · ml^−1^ in pyridine) and incubated in an oven (MKX-J1-10, Qingdao Makewave Microwave Technology Co. Ltd., Qingdao, China) at 37 °C for 2 h. Subsequently, samples were derivatized with trimethylsilylation containing trimethylchlorosilane 70 °C for 1 h [31]. After the temperature of samples were fell to room temperature, GC-TOF/MS analysis was performed using an Agilent 7890 gas chromatograph system (California, USA) coupled with a capillary DB-5MS GC/MS columns. A 1-μl aliquot of the analyte was injected in splitless mode. As the carrier gas, helium was used, with a flow rate of 1 mL · min^−1^ after the front inlet purge flow was 3 mL · min^−1^. The column temperature was maintained at 90 °C during the first 0.25 min; further, the temperature was increased to 180 °C at a rate of 10 °C min^−1^ and to 240 °C at a rate of 5 °C min^−1^. The injector temperature was 280 °C and transfer line 280 was used. Ionization in the ion source at a temperature of 220 °C was coupled with the electron energy of 70 eV. Mass spectra were recorded in the range 20–600 m · z^−1^ at a rate of 100 spectra per second. The whole analysis time was 35 min.

### Statistical analysis

SPSS v. 13 was used for the statistical analyses of the data for the plant growth, *P*_n_, *g*_s_ and *E* and variations of metal element contents, as well as for evaluation of the statistical significance and correlations. All treatments were replicated five times, and the data obtained were expressed as means and standard errors. Means followed by different letters in the same stress type are significantly different at *P* < 0.01 according to Duncan’s method. Identification of metabolites was performed by searches in FiehnLib and the commercial EI-MS libraries [32]. Then, at least 80% of missing values were removed and replaced with a small value, which was half of the minimum positive value in the original data. The data were filtered using the interquantile range, and the total mass of the signal integration area was normalized for each sample. SIMCA-P 14.0 software package (Umetrics, Umea, Sweden) was employed to run the principal component analysis (PCA) and projections to latent structure-discriminant analysis (PLS-DA). In addition, metabolite pathways were searched on non-commercial databases, such as KEGG (http://www.genome.jp/kegg/) and MetaboAnalyst website (www.metaboanalyst.ca/) [33].

## Results

### Growth status of maize seedlings under neutral salt and alkaline salt stresses

**Fig. 1:**
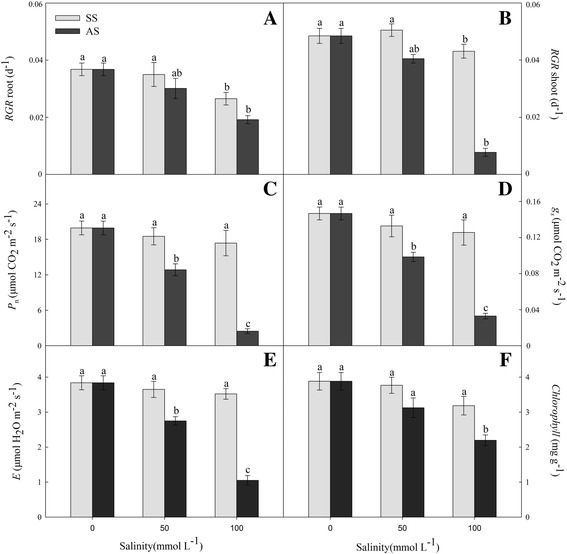
Effects of neutral salt stress (SS) and alkaline salt stress (AS) on the relative growth rate (*RGR*) of root (**a**) and shoot (**b**); net photosynthetic rate (*P*_N_) (**c**), stomatal conductance (*g*_s_) (**d**), transpiration rate (*E*) (**e**), and total of chlorophyll content (TCC) (**f**) of maize. Values represent the means of five replicates. Means followed by different letters in the same stress type are significantly different at *P* < 0.01 according to Duncan’s multiple range test

Distinct changes in the relative growth ratio (RGR) values were exhibited by the maize seedlings in response to their 15-day exposures to neutral salt and alkaline salt stresses. No significant effect was observed in the *RGR* levels of the roots and shoots of the treatment groups under 50 mM neutral salt stress as compared to the control group. However, the neutral salt stress at 100 mM suppressed substantially the growth of maize roots and shoots (Fig. 1a and b, *P* < 0.01). Alkaline salt stress significantly reduced *RGR* values in the shoots and roots, and the reductions at 100 mM were more appreciable than those at 50 mM (Fig. 1a and b, *P* < 0.01). The results showed that slight changes in the photosynthetic indices and chlorophyll contents were observed under neutral salt stress as compared with the control group; by contrast, significant reductions of their values were caused by the different levels of alkaline salt stress (Fig. 1c–f; *P* < 0.01).

### Metal elements

After the treatment with neutral salt and alkaline salt stresses, Na and K were the major metal elements found in the maize root and shoot tissues. The Na levels increased, while the levels of K decreased. However, these changes were greater under alkaline salt stress than under neutral salt stress (Table 2, *P* < 0.01). With increasing the stress concentrations, under neutral salt stress, the maize seedlings showed a significant decrease in Ca level in both the root and shoot; however, the Ca level increased dramatically under alkaline salt stress (Table 2, *P* < 0.01). Neutral salt stress caused only slight changes in the level of Mg in the shoot, which was in contrast to its significant decrease in the root of the plants subjected to alkaline salt stress (Table 2, *P* < 0.01). The metal elements concentrations of Fe, Cu, and Zn in the root were not affected by either of the stress conditions. The level of Cu increased significantly under high alkaline salt stress, while Fe and Zn levels decreased (Table 2, *P* < 0.01). Mn level was enhanced under both stress conditions, but higher elevation was found under neutral salt stress than under alkali stress (Table 2, *P* < 0.01). The level of B was not substantially affected by the two stresses induced.

**Table 2:** Elemental contents (μmol/g DW) in the roots and shoots of maize plants under 50 and 100 mM neutral salt stress and alkali stress

Tissue	Treatment	Salinity (mM)	Na	K	Ca	Mg	Fe	Cu	Zn	Mn	B
Roots	CK	0	515.46 ± 23.73^c^	182.00 ± 8.06^a^	113.48 ± 7.65^c^	44.41 ± 2.06^a^	5.01 ± 0.52^a^	1.77 ± 0.06^a^	0.13 ± 0.00^a^	0.22 ± 0.01^b^	0.23 ± 0.01^b^
	SS	50	688.43 ± 36.51^b,c^	191.53 ± 10.69^a^	101.25 ± 5.05^c,d^	40.58 ± 2.81^a^	4.57 ± 0.32^a^	1.18 ± 0.06^a^	0.12 ± 0.00^a^	0.28 ± 0.00^b^	0.20 ± 0.00^b^
		100	1007.20 ± 53.67^b^	155.86 ± 9.73^a,b^	88.56 ± 8.42^d^	45.28 ± 3.24^a^	5.96 ± 0.09^a^	1.36 ± 0.08^a^	0.13 ± 0.00^a^	0.48 ± 0.02^a^	0.27 ± 0.00^b^
	AS	50	917.56 ± 40.88^b^	130.45 ± 10.33^b^	137.09 ± 4.68^b^	38.87 ± 4.02^a,b^	4.11 ± 0.11^a^	1.86 ± 0.10^a^	0.14 ± 0.00^a^	0.31 ± 0.01^a,b^	0.26 ± 0.03^b^
		100	1303.69 ± 90.81^a^	101.14 ± 5.98^c^	162.56 ± 12.44^a^	28.07 ± 1.50^b^	5.77 ± 0.22^a^	2.13 ± 0.03^a^	0.13 ± 0.01^a^	0.37 ± 0.03^a,b^	0.52 ± 0.02^a^
Shoots	CK	0	70.47 ± 5.14^c^	586.19 ± 35.60^a^	65.17 ± 5.35^c^	87.80 ± 5.49^a^	5.56 ± 0.17^a^	0.27 ± 0.01^b^	0.16 ± 0.00^a^	0.32 ± 0.01^b^	0.82 ± 0.06^a^
	SS	50	89.05 ± 7.39^b,c^	516.88 ± 25.00^a^	62.87 ± 5.16^c^	80.48 ± 6.02^a^	4.75 ± 0.24^a^	0.26 ± 0.01^b^	0.17 ± 0.00^a^	0.35 ± 0.01^b^	0.78 ± 0.04^a^
		100	144.66 ± 9.51^b^	429.19 ± 30.17^a,b^	60.02 ± 2.99^d^	73.73 ± 2.43^a^	3.24 ± 0.09^a,b^	0.18 ± 0.00^b^	0.10 ± 0.00^a,b^	0.68 ± 0.01^a^	1.04 ± 0.07^a^
	AS	50	148.73 ± 6.54^b^	462.58 ± 20.11^a,b^	78.85 ± 7.01^b^	76.98 ± 4.56^a^	4.21 ± 0.13^a^	0.32 ± 0.02^b^	0.13 ± 0.00^a^	0.36 ± 0.00^b^	0.89 ± 0.04^a^
		100	194.36 ± 12.78^a^	294.97 ± 16.85^b^	109.45 ± 9.25^a^	76.97 ± 3.48^a^	2.40 ± 0.21^b^	0.50 ± 0.01^a^	0.09 ± 0.00^b^	0.50 ± 0.01^a,b^	0.94 ± 0.03^a^

Values represent the means of five replicates. Means followed by different letters in the same stress type are significantly different at *P* < 0.01 according to Duncan’s method

### Metabolic trajectory for high salinity-induced responses of maize

**Fig. 2:**
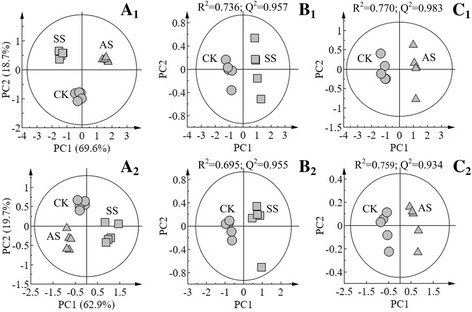
Score plots of the principal component analysis displaying the metabolomic trajectory of the root (*A*_*1*_) and shoot (*A*_*2*_) of maize seedlings under no salinity stress (CK), neutral salt stress (SS), and alkaline salt stress (AS). Scores obtained by orthogonal partial least-squares discriminant analysis (PLS-DA) exhibiting the dependence of the effects of neutral salt stress on maize on salinity levels: CK vs. SS in roots (*B*_*1*_) and shoots (*B*_*2*_); CK vs. AS in (*C*_*1*_) roots and shoots (*C*_*2*_)

In this study, 44 kinds of metabolites were identified and their concentrations were determined under normal and salinity stresses conditions. The scores plot of PCA results showed that approximately 70% and 63% variability in the three groups of samples can be explained using two principal components in the roots and shoots, respectively (Fig. 2, A_1_ and A_2_). In addition, we performed pairwise comparison of the data obtained by PLS-DA, whose score plot exhibited an obvious distinction between the tissues of maize treated with salt or alkaline salt stress for 15 days with good model quality (Fig. 2, B_1_–C_2_).

Our results showed that neutral salt stress exerted strongly negative effects on glycolysis in the roots, as shown by reduction in the levels of glucose, glucose-6-P, fructose-6-P and 3PGA. Neutral salt stress induced an increase in the content of sugars, including fructose, sucrose, talose, and myo-inositol. Meanwhile, the levels of most amino acids such as glutamate, alanine, serine, and valine, were significantly decreased (Table 3). Under neutral salt stress, the levels of shikimic acid, quinic acid, chlorogenic acid, and ferulic acid, which participate in the *shikimic* pathway, were reduced significantly as compared to those of the control (Table 3). The *TCA* cycle was not significantly affected by neutral salt stress, but it was enhanced under alkaline salt stress causes increasing the levels of citric acid, aconitic acid, succinic acid, fumaric acid and malic acid (Table 3). In addition, cinnamic and ferulic acid levels were also promoted by the alkaline salt stress treatment (Table 3). Nevertheless, under alkaline salt stress, the levels of some sugars and their derivatives, including sucrose, glucose, raffinose, and galactinol, as well as those of certain amino acids, such as glumate, alanine, valine and serine, were reduced substantially (Table 3). In addition, significant accumulation of proline in the roots was observed under both stresses (Table 3). The two stresses did not remarkably affect the levels of fatty acids in the root tissues (Table 3).

**Table 3:** Relative concentration and fold-changes in the levels of major metabolites in roots and shoots of maize seedlings after 15 d of salt and alkaline treatments. Fold-changes were calculated using the formula log_2_^(treatment/control)^

Metabolic pathways	Metabolites name	Major metabolites in roots					Major metabolites in shoots				
		Relative concentration			Fold changes		Relative concentration			Fold changes	
		CK	SS	AS	Log_2_^(SS/CK)^	Log_2_^(AS/CK)^	CK	SS	AS	Log_2_^(SS/CK)^	Log_2_^(AS/CK)^
*TCA* cycle	Citric acid	46.97 ± 3.85	32.51 ± 2.26	94.78 ± 5.39	−0.53	1.01^*^	3.66 ± 0.38	1.99 ± 0.10	5.24 ± 0.62	−0.88^*^	0.52
	Aconitic acid	68.74 ± 4.73	80.81 ± 5.04	136.35 ± 8.75	0.23	0.99^*^	0.50 ± 0.03	0.36 ± 0.02	0.83 ± 0.06	−0.47	0.73
	α-Ketoglutaric acid	0.31 ± 0.02	0.20 ± 0.00	0.26 ± 0.01	−0.62	−0.27	1.48 ± 0.04	0.42 ± 0.01	0.97 ± 0.05	−1.82^*^	−0.60
	Succinic acid	7.60 ± 0.83	10.58 ± 0.29	41.12 ± 3.06	0.48	2.44^*^	4.59 ± 0.27	2.76 ± 0.38	3.29 ± 0.64	−0.73	−0.48
	Fumaric acid	1.09 ± 0.04	0.83 ± 0.01	3.08 ± 0.19	−0.39	1.51^*^	0.50 ± 0.04	0.36 ± 0.02	0.44 ± 0.07	−0.48	−0.20
	Malic acid	9.90 ± 0.95	6.76 ± 0.38	22.15 ± 1.75	−0.55	1.16^*^	66.10 ± 5.03	39.87 ± 2.36	45.92 ± 4.69	−0.73^*^	−0.53
*Glycolysis*	Glucose	1.58 ± 0.09	0.37 ± 0.01	0.28 ± 0.00	−2.11^*^	−2.49^*^	0.06 ± 0.00	0.07 ± 0.00	0.04 ± 0.00	0.23	−0.65
	Glucose-6-P	0.81 ± 0.03	0.18 ± 0.00	0.68 ± 0.05	−2.15^*^	−0.26	0.16 ± 0.00	0.46 ± 0.03	0.06 ± 0.00	1.48^*^	−1.40^*^
	Fructose-6-P	0.53 ± 0.07	0.12 ± 0.00	0.43 ± 0.15	−2.08^*^	−0.28	0.14 ± 0.00	0.61 ± 0.03	0.10 ± 0.00	2.11^*^	−0.50
	3-Phosphoglycerate	0.34 ± 0.01	0.15 ± 0.00	0.19 ± 0.00	−1.14^*^	−0.85	0.03 ± 0.00	0.07 ± 0.00	0.02 ± 0.00	1.03^*^	−1.05^*^
	Phosphoenolpyruvate	0.03 ± 0.00	0.03 ± 0.00	0.02 ± 0.00	0.20	−0.34	0.01 ± 0.00	0.05 ± .0.00	0.01 ± .0.00	2.06^*^	−0.46
	Pyruvate	0.37 ± 0.01	0.53 ± 0.03	0.66 ± 0.07	0.53	0.85	5.47 ± 0.35	4.35 ± 0.12	0.51 ± 0.04	−0.33	−3.42^*^
*Shikimic path way*	Shikimic acid	3.77 ± 0.15	1.20 ± 0.07	2.76 ± 0.31	−1.65^*^	−0.45	3.53 ± 0.24	4.81 ± 0.42	1.32 ± 0.09	0.44	−1.42^*^
	Quinic acid	7.11 ± 0.53	1.14 ± 0.04	4.36 ± 0.18	−2.64^*^	−0.71	4.41 ± 0.32	4.52 ± 0.13	1.55 ± 0.05	0.04	−1.51^*^
	Chlorogenic acid	0.25 ± 0.03	0.01 ± 0.00	0.20 ± 0.00	−4.49^*^	−0.35	3.02 ± 0.21	5.93 ± 0.56	5.70 ± 0.28	0.97	0.92
	Cinnamic acid	0.86 ± 0.06	0.54 ± 0.01	14.00 ± 0.65	−0.67	4.03^*^	2.68 ± 0.31	4.32 ± 0.12	10.04 ± 0.59	0.69	1.90^*^
	Ferulic acid	0.14 ± 0.00	0.04 ± 0.00	0.34 ± 0.01	−1.88^*^	1.24^*^	0.22 ± 0.01	0.41 ± 0.02	0.27 ± 0.01	0.89	0.31
Amino acids	Glutamate	55.50 ± 4.98	21.19 ± 0.99	92.51 ± 6.35	−1.39^*^	0.74	10.55 ± 0.27	2.04 ± 0.23	3.97 ± 0.31	−2.37^*^	−1.41^*^
	Alanine	53.01 ± 3.56	4.73 ± 0.32	64.02 ± 4.01	−3.49^*^	0.27	33.98 ± 1.99	70.78 ± 5.39	7.29 ± 0.51	1.06^*^	−2.22^*^
	γ-Aminobutyric acid	2.77 ± 0.15	3.36 ± 0.27	2.65 ± 0.19	0.28	−0.06	0.41 ± 0.02	1.26 ± 0.73	0.25 ± 0.03	1.61^*^	−0.74
	Valine	11.35 ± 0.75	2.70 ± 0.31	11.70 ± 0.85	−2.07^*^	0.04	0.74 ± 0.03	1.26 ± 0.07	0.85 ± 0.02	0.78	0.21
	Serine	18.16 ± 1.07	6.67 ± 0.35	15.40 ± 0.87	−1.44^*^	−0.24	2.61 ± 0.31	11.42 ± 0.75	3.70 ± 0.18	2.13^*^	0.50
	Aspartate	4.20 ± 0.12	2.34 ± 0.17	3.20 ± 0.20	−0.84^*^	−0.39	1.09 ± 0.04	0.56 ± 0.03	0.39 ± 0.00	−1.46^*^	−0.51
	Asparagine	11.14 ± 0.61	14.11 ± 0.57	11.65 ± 0.77	0.34	0.06	0.01 ± 0.00	0.02 ± 0.00	0.01 ± 0.00	1.04^*^	−0.84
	Threonine	6.24 ± 0.12	2.14 ± 0.10	5.42 ± 0.36	−1.54^*^	−0.20	0.76 ± 0.05	1.64 ± 0.19	0.39 ± 0.03	1.10^*^	−0.99^*^
	Proline	0.60 ± 0.08	8.38 ± 0.69	1.58 ± 0.09	3.80^*^	1.40^*^	0.33 ± 0.03	19.21 ± 1.14	0.82 ± 0.06	5.86^*^	1.32^*^
	Isoleucine	5.66 ± 0.35	2.52 ± 0.16	5.21 ± 0.31	−1.17^*^	−0.12	0.16 ± 0.00	0.35 ± 0.03	0.22 ± 0.02	1.08^*^	0.46
	Glycine	8.97 ± 0.59	4.09 ± 0.37	5.00 ± 0.29	−1.13^*^	−0.84	4.77 ± 0.25	6.33 ± 0.65	6.00 ± 0.54	0.41	0.33
	Leucine	7.06 ± 0.53	1.59 ± 0.10	4.82 ± 0.21	−2.15^*^	−0.55	0.24 ± 0.06	0.15 ± 0.00	0.36 ± 0.03	−0.68	0.62
	Glutamine	0.43 ± 0.15	0.01 ± 0.00	0.33 ± 0.02	−5.68^*^	−0.41	0.05 ± 0.00	0.03 ± 0.00	0.04 ± 0.00	−0.56	−0.41
	Methionine	0.98 ± 0.05	0.37 ± 0.01	0.74 ± 0.02	−1.39^*^	−0.41	0.03 ± 0.00	0.03 ± 0.00	0.01 ± 0.00	−0.04	−1.30^*^
	Phenylalanine	0.35 ± 0.02	0.07 ± 0.00	0.48 ± 0.04	−2.25^*^	0.47	0.02 ± 0.00	0.08 ± 0.00	0.04 ± 0.00	2.04^*^	0.84
Sugars	Fructose	104.07 ± 8.35	176.79 ± 7.69	118.77 ± 6.51	0.76^*^	0.19	33.13 ± 2.87	48.83 ± 2.13	36.26 ± 2.00	0.56	0.13
	Sucrose	7.29 ± 0.64	22.18 ± 2.09	1.34 ± 0.07	1.60^*^	−2.45^*^	0.12 ± 0.00	0.12 ± 0.00	0.12 ± 0.01	−0.05	−0.07
	Talose	0.92 ± 0.06	2.03 ± 0.10	0.47 ± 0.03	1.14^*^	−0.97	26.62 ± 2.09	40.49 ± 1.57	29.32 ± 1.64	0.61	0.14
	Raffinose	0.49 ± 0.04	0.76 ± 0.03	0.23 ± 0.01	0.64	−1.11^*^	2.81 ± 0.19	7.34 ± 0.67	3.90 ± 0.25	1.38^*^	0.47
	myo-Inositol	5.48 ± 0.47	13.22 ± 0.96	9.59 ± 0.59	1.27^*^	0.81	27.91 ± 2.13	41.22 ± 3.61	15.01 ± 0.57	0.56	−0.89
	Ribose	2.11 ± 0.15	2.84 ± 0.20	2.53 ± 0.11	0.43	0.27	3.35 ± 0.17	5.07 ± 0.35	1.11 ± 0.04	0.60	−1.60^*^
	Galactinol	3.05 ± 0.19	3.30 ± 0.56	1.48 ± 0.10	0.11	−1.05^*^	3.07 ± 0.13	10.59 ± 0.63	5.49 ± 0.45	1.79^*^	0.84
Fatty acids	Palmitic acid	4.65 ± 0.32	5.62 ± 0.71	6.65 ± 0.43	0.27	0.52	18.69 ± 1.03	8.83 ± 0.51	34.71 ± 2.17	−1.08^*^	0.89^*^
	Stearic acid	1.35 ± 0.07	1.24 ± 0.07	1.68 ± 0.08	−0.13	0.31	2.28 ± 0.14	4.12 ± 0.34	1.95 ± 0.10	0.85	−0.23
	Arachidic acid	0.15 ± 0.00	0.11 ± 0.00	0.17 ± 0.00	−0.43	0.22	0.38 ± 0.01	0.30 ± 0.01	0.41 ± 0.02	−0.34	0.12
	Linoleic acid	0.28 ± 0.03	0.17 ± 0.00	0.32 ± 0.02	−0.75	0.19	0.13 ± 0.00	0.07 ± 0.00	0.26 ± 0.01	−0.82	1.01^*^
	Oleic acid	0.22 ± 0.02	0.15 ± 0.00	0.23 ± 0.00	−0.59	0.07	0.04 ± 0.00	0.02 ± 0.00	0.09 ± 0.00	−1.10^*^	1.18^*^

*indicates significance (*P* < 0.01)

In the shoots, the lower citric acid, α-ketoglutaric acid, and malic acid levels indicated that neutral salt stress had inhibited the *TCA* cycle; however, it was not significantly affected by alkaline salt stress (Table 3). Glucose-6-P, fructose-6-P, 3PGA, and PEP levels were dramatically increased. Furthermore, the contents of raffinose and galactinol, which are associated with glycolysis, were enhanced under neutral salt stress (Table 3). Meanwhile, glycolysis was significantly inhibited under alkaline salt stress, causing significant reductions in the levels of glucose-6-P, 3PGA, and pyruvate (Table 3). Neutral salt stress had an insignificant effect on the *shikimate* pathway, but alkaline salt stress inhibited *shikimate* pathway and significantly decreased the levels of shikimic and quinic acids (Table 3). Neutral salt stress dramatically enhanced the levels of amino acids in the shoots, including those of alanine, GABA, serine, asparagine, threonine, isoleucine, and phenylalanine. On the other hand, glutamate and aspartate (used for proline synthesis) were depleted dramatically probably due to synthesis of proline metabolites (Table 3). Under alkaline salt stress, the biosynthesis of amino acids, including glutamate, alanine, threonine, and methionine, was considerably inhibited (Table 3). A significant, 5.86-fold increase in the level of proline was established in the neutral salt stress treatment, whereas its content was 1.32-fold higher under alkaline salt stress that that in the control (Table 3). In addition, alkaline salt stress significantly decreased ribose levels in the shoots (Table 3). The contents of palmitic and oleic acid, which belong to the group of the fatty acids, decreased under neutral salt stress, while their levels increased under alkaline salt stress (Table 3).

## Discussion

### Growth, photosynthesis parameters and pigment content

During their seedling stage, plants are sensitive to adverse external factors; therefore, seedlings stage is the optimum time to research plants abiotic tolerance [33]. *RGR* could reflect the growth conditions of a plant and is considered as an important index in determining the degree of stress of plants. Salinity generally inhibits plants growth and even leads to death [15]. Our findings evidenced the adverse effect of alkaline salt stress on root growth (Fig. 1a and b). These observations indicated that although the impacts of neutral salt and alkaline salt stresses are similar to a certain extent, they are actually two distinct kinds of stresses. The additional impact of high-pH stress under high pH conditions contributes to achieving even more pronounced harmful effects than the ones caused by salinity stress [15, 16].

To obtain insights into the mechanisms involved and the nature of stress-induced damage to the photosynthetic apparatus, we also examined the changes in the photosynthesis and pigment content, which are parameters of stress as reported earlier [16]. Moderate levels of neutral salt stress had a little impact on the major parameters of photosynthesis, whereas alkaline salt stress exerted a more severe adverse influence, leading to a decline in *P*_n_, *g*_s_ and *E* (Fig. 1c–e, *P* < 0.01). In addition, the chlorophyll content was not diminished in the neutral salt stress treatment, but it declined sharply under the conditions of alkaline salt stress (Fig. 1f, *P* < 0.01). It is well known that plant species have three metabolism processes response to massive Na^+^ under salt stress, including exclusion, compartmentalization and ion transport [34]. The Na^+^ exclusion mechanism dependent on a Na^+^/H^+^ antiport, such as salt overly sensitive 1 type (*SOS*1), and the transmembrane proton gradient (H^+^-ATPase) decided to exchange activity of Na^+^ and H^+^ [23]. The high pH value decreased external protons and weakens the exchange activity of the Na^+^/H^+^ antiport, causing the exclusion of Na^+^ has been inhibited and enhancing Na^+^ accumulated in vivo under alkaline salt stress [34, 35]. The negative action of alkaline salt stress on photosynthetic capacity and chlorophyll content was probably due to the accumulation of Na^+^ in the cytoplasm as well as to the destruction of the structure and suppression of the functions of chloroplasts [16, 35]. Superfluous Na^+^ and high pH value affect Fe accumulation; which is known to play important role in chlorophyll biosynthesis and photosynthetic rate in plants. The content of Fe decreased caused great decrease in content of chlorophyll content, photosynthesis and therefore a decreased in biomass [36–38].

### Metal elements

The cytoplasm of higher plants normally maintains high K^+^ and low Na^+^ concentrations to facilitate the proper functioning of many enzymes and the normal action of the catalyzed by them important physiological processes; osmotic regulation is the main mechanism to sustain this state [1, 23]. The findings of this investigation confirm that competitive relationships exist between K and Na during their uptake under the conditions of high salt and alkalinity stress; the amount of Na increased, while the total K content decreased. These effects were more pronounced under alkaline salt stress than under neutral salt stress. Maize plants respond to the stress caused by high pH by a considerable increase in the accumulation of Na and Ca in their tissues, a reaction that does not occur under neutral salt stress. A large number of plants possess a remarkable mechanism for exclusion of Na^+^ that is dependent on the gradient of H^+^ across the cell membranes of the roots [34]. For example, in the model plant *Arabidopsis*, *SOS*1 protein has been identified that it functions in exclusion of Na^+^ from epidermal cells of roots to the rhizosphere, which may play an important role in retrieving Na^+^ from roots to shoots under salt stress, so this phenomenon might be the basis metabolism response to alkaline injury [34]. In addition, the research found that Ca^2+^ plays important roles in the regulating AtSOS3–AtSOS2 protein kinase pathway mediates expression, and it also responsive AtNHX and AtSOS1 protein regulation activities of Na^+^ transporters, which indicated that Ca^2+^ being the key signal component in the *SOS* system in *Arabidopsis* and some other plant species [23, 34]. In conclusion, we infer that by excluding Na^+^ and Ca^2+^ play important roles in plant alkaline tolerance. In this study, neutral salt stress reduced Ca^2+^ accumulation in maize roots, but alkaline salt stress strongly enhanced its accumulation in the shoots and roots. The increase of Ca^2+^ level in tissues of maize seedlings during alkaline salt stress can instantly activate the *SOS*–Na^+^ system for exclusion and diminish the damage to the plants caused by Na^+^ toxicity.

### Neutral salt and alkaline salt stresses responses in maize metabolism

The excessive concentration of Na^+^ and the osmotic stress caused by high salinity have adverse impacts on the functions of the roots, inducing the generation of reactive oxygen species, such as H_2_O_2_ and O_2_^3 −^, and causing intracellular hyper-ammonia stress [34, 39]. Under saline conditions, to decrease the water potential of the cytoplasm to prevent it from dehydration, plants usually accumulate organic solutes in their vacuoles, such as betaine, proline, free sugars, and polyalcohol [11, 34].

The results indicated that the GC-MS metabonomic analysis is an excellent method for understanding the molecular responses to salinity, which could reflect the integration of genomics, proteomics, transcriptomics and other different regulatory processes [40]. By protecting plant cell membranes and proteins and by functioning as a scavenger of reactive oxygen species, proline plays an important role in the response of plants to neutral salt stress [41, 42]. In the present examination, we detected dramatically elevated levels of proline in both the roots and shoots, which contributed significantly to the osmotic regulation in the experimental maize plants subjected to neutral salt stress. Similar findings on this protective function of proline were obtained by Wu et al. [43] and Yang et al. [16]. The neutral salt stress-induced elevation of glutamate levels indicates that proline biosynthesis is important for the control of salinity-induced osmotic pressure. However, the level of proline accumulation was significantly lower under alkaline salt stress than under neutral salt stress. Our results imply that the high pH values under alkaline salt stress conditions might suppress the activity of Δ1-pyrroline-5-carboxylate synthetase (P5CS), inhibiting the conversion of glutamate into proline.

The concentrations of sugars, such as glucose, fructose, and sucrose, have been found to increase in response to neutral salt stress [16, 44]. Our results showed that the levels of fructose, sucrose, talose, and myo-inositol in the roots, as well as those of raffinose and galactinol in the shoots, were dramatically increased in the maize plants under neutral salt stress, but glucose showed decreased trend (Table 3). In Gavaghan et al. [45] study, it confirmed that sucrose was increased significantly while glucose decreased in roots of maize under salt stress using nuclear magnetic resonance (NMR) spectroscopy. In plants, sugars are commonly produced by photosynthesis, degradation of polysaccharides, and gluconeogenesis [44]. In our investigation, we found that the photosynthetic rate of the seedlings subjected to neutral salt stress was similar to that of the ones in the control group. This result suggests that the process of gluconeogenesis was enhanced in the plants under neutral salt stress, implying that degradation of polysaccharides, used as a carbon source, was probably promoted to achieve maintenance of osmotic balance (Fig. 1 and Table 3). Nevertheless, the concentrations of glucose, sucrose, and ribose were significantly reduced in the experimental maize seedlings in response to alkaline salt stress. The rate of photosynthesis was substantially decreased by alkaline salt stress, resulting in inhibited production of reducing forces and limited N metabolism, which in turn lowered sugar production (Fig. 1 and Table 3). The toxic levels of Na^+^ that had accumulated in plant cells at high pH values might have also had detrimental effects on sugar production.

**Fig. 3:**
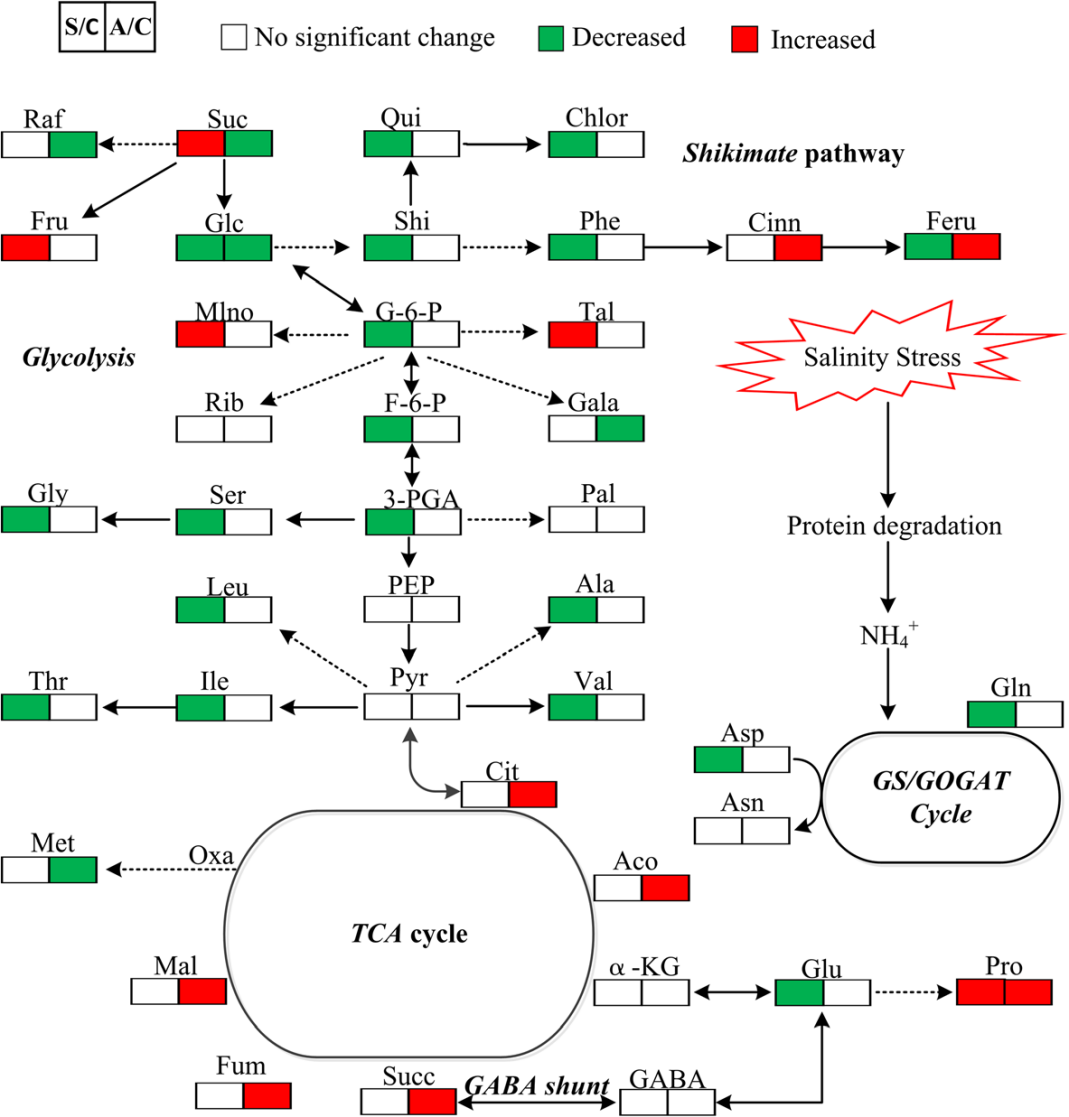
Changes in the metabolism pathways in the roots of maize seedlings after 15-d treatment with alkaline salt stress. Suggested changes in the metabolic network in maize seedlings under alkali stress. Data obtained by PLS-DA. *Red boxes* refer to significantly enhanced metabolites, whereas those in *green boxes* were significantly reduced (*P* < 0.01)

**Fig. 4:**
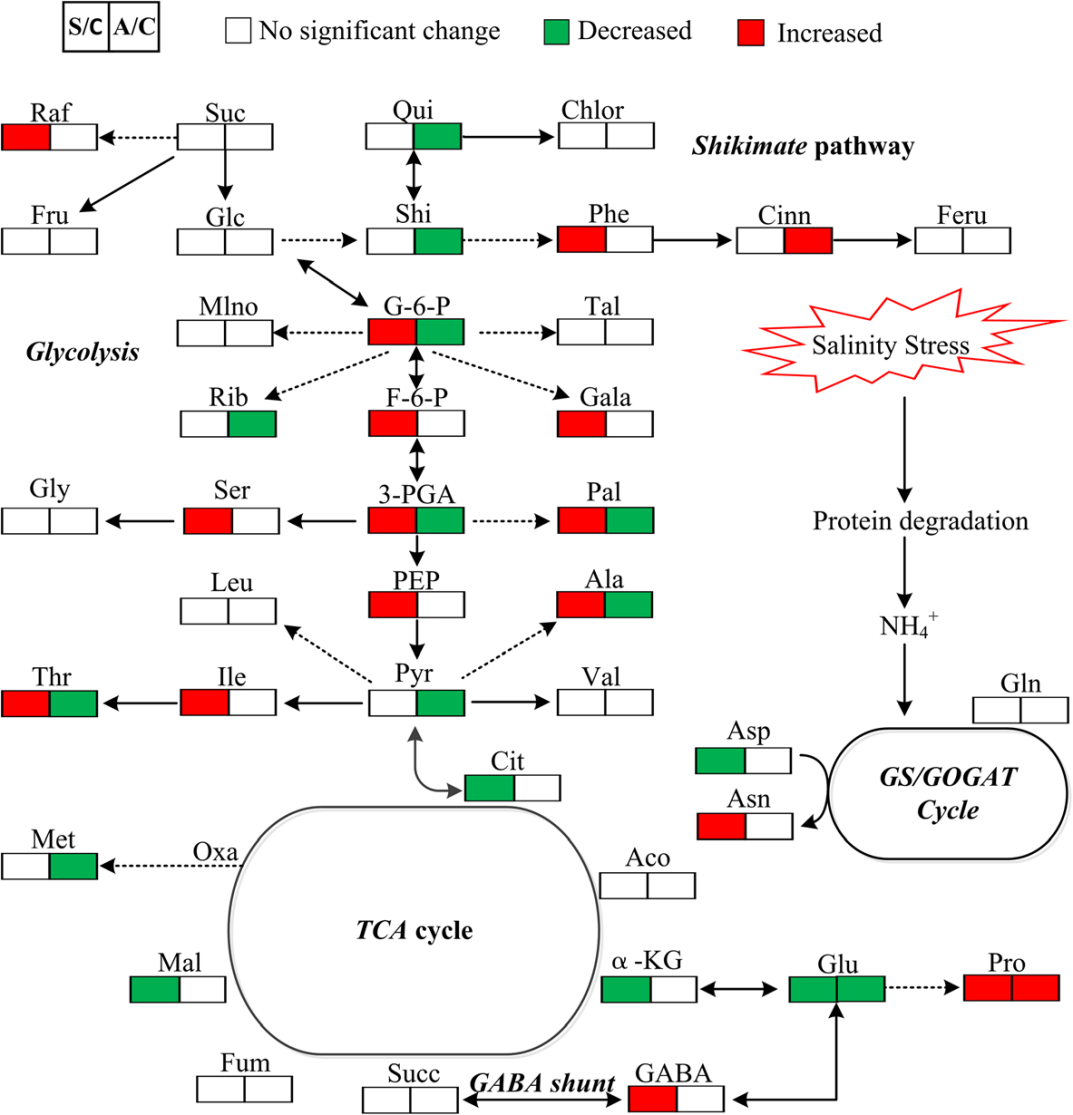
Changes in the metabolism pathways in the shoots of maize seedlings after 15-d treatment with alkaline salt stress. Suggested changes in the metabolic network in maize seedlings under alkaline salt stress, as obtained through PLS-DA. *Red boxes* denote significantly enhanced metabolites, whereas those in *green boxes* were significantly reduced (*P* < 0.01)

In the present study, evident differences between the responses to neutral salt and alkaline salt stresses were found in the content of metabolites in the investigated maize plants. Neutral salt stress stimulated sugar accumulation, but glycolysis, the *shikimic* pathway, and amino acid synthesis in roots, were inhibited (Fig. 3). By contrast, glycolysis and the synthesis of amino acids and fatty acids in shoots were enhanced, while the *TCA* cycle was suppressed (Fig. 4). These results indicate that under neutral salt stress, the most important compatible solutes are the sugars in the roots and that active synthesis metabolism is a basic response of shoots in developing their tolerance to neutral salt stress. The increased levels of serine, isoleucine, and phenylalanine in shoots were probably related to glycolysis as a way of relieving transamination products because they are glucogenic amino acids (Fig. 3). Fatty acids maybe an important compatible solute in shoots of maize plants subjected to neutral salt stress, especially palmitic acid and oleic acid

Under alkaline salt stress, the *TCA* cycle, *shikimic* pathway, and organic accumulation were enhanced significantly; however, the synthesis of amino acids was inhibited significantly in the roots. Furthermore, a decrease in the content of glutamate and alanine indicated that the accumulation of these amino acids enhanced *GABA* shut biosynthesis process, leading to increases in the *TCA* cycle (Fig. 3). Under alkaline salt stress, the glycolysis and synthesis of amino acids and fatty acids in shoots were inhibited (Fig. 4). These results indicated that energy and high levels of organic acids are the key adaptive mechanisms by which maize seedlings maintain their intracellular ion homeostasis under alkaline salt stress. The accumulation of organic acids in vacuoles might play a central role in the regulation of intracellular pH through neutralization of excess cations [15, 16]. Excessive Na^+^ ion concentrations may induce a cascade of signal transduction events which culminate in the promotion of the synthesis of organic acid leading to the negative charge deficit in maize. Consequently, accumulation of various organic acids in plant cells is necessary.

The reduction in amino acid levels in maize tissues induced by high pH could be attributed to the decrease of N metabolism rates. To realize absorption of nutrients, such as nitrates (NO_3_^−^) and ammonium (NH_4_^+^), the roots of plants utilize a number of transport systems [17]. For instance, the members of AMT protein family perform transportation of NH_4_^+^, while the representatives of the NRT protein family realize the transport of NO_3_^−^. NRT regulates the uptake of NO_3_^−^, whereas AMT controls the absorption of NH_4_^+^ possibly through the transmembrane proton gradient [46]. The absence of an external for the plasma membrane of the roots supply of protons under alkaline salt stress conditions might retard the actions of NRT and AMT, leading to a decrease in the uptake of NO_3_^−^ and NH_4_^+^. This phenomenon might influence nearly all processes of plant metabolism, which was confirmed by our findings. At high pH values, alkaline salt stress considerably suppressed the rate of photosynthesis, leading to a decline in glycolysis, reduced production of sugars and amino acid, and limited N metabolism. Consequently, we speculate that high concentrations of organic acids and energy are potential major factors whose action is required for the adaptation of maize plants, achieving proper support of the balance of intracellular ion concentrations and exerting control on high pH values under high alkaline salt stress.

## Conclusion

Alkaline salt stress suppressed more considerably the photosynthesis and growth of maize than neutral salt stress. Moreover, under alkaline salt stress, metal ions formed massive precipitates that reduced plant nutrient availability. On the other hand, high salinity induced metabolic changes in gluconeogenesis; enhanced formation of sugars was established probably as a reaction to attenuate the osmotic stress caused by neutral salt stress. The active synthesis of amino acids in shoots was essential to the development of salt tolerance. However, alkaline salt stress conditions suppressed substantially the levels of N metabolism, glycolysis, and the production of sugars and amino acids. Our findings suggest the presence of different mechanisms involves in plant responses to neutral salt and alkaline salt stresses. The increased concentration of organic acids and the enhanced metabolic energy might be major factors contributing to the maintenance of intracellular ion balance in maize plants and counteract the negative effects of high pH under alkaline salt stress.

## Abbreviations

3-PGA: 3-phosphoglycerate
Aco: Aconitic acid
Ala: Alanine
Asn: Asparagine
Asp: Aspartic acid
Cit: Citric acid
F6P: Fructose-6-phosphate
Fru: Fructose
Fum: Fumaric acid
G6P: Glucose-6-phosphate
GABA: γ-aminobutyric acid
Gala: Galactinol
Glc: Glucose
Gln: Glutamine
Glu: Glutamate
Gly: Glycine
Hep: Heptulose
Ile: Isoleucine
Kes: Kestose
Leu: Leucine
Mal: Malic acid
mIno: myo-Inositol
PEP: Phosphoenolpyruvate
Phe: Phenylalanine
Pro: Proline
Pyr: Pyruvate
Raf: Raffinose
Rib: Ribose
Ser: Serine
Sor: Sorbitol
Suc: Sucrose
Succ: Succinic acid
Tal: Talose
Thr: Threonine
Val: Valine
α-KG: α-ketoglutaric acid
